# Right versus left ventricular remodeling in heart failure due to chronic volume overload

**DOI:** 10.1038/s41598-021-96618-8

**Published:** 2021-08-24

**Authors:** Tereza Havlenova, Petra Skaroupkova, Matus Miklovic, Matej Behounek, Martin Chmel, Dagmar Jarkovska, Jitka Sviglerova, Milan Stengl, Michal Kolar, Jiri Novotny, Jan Benes, Ludek Cervenka, Jiri Petrak, Vojtech Melenovsky

**Affiliations:** 1 Department of Cardiology, Institute for Clinical and Experimental Medicine - IKEM, Videnska 1958/9, 140 21 Prague 4, Czech Republic; 2 Department of Pathophysiology, Second Faculty of Medicine, Charles University, Prague, Czech Republic; 3 BIOCEV, First Faculty of Medicine, Charles University, Prague, Czech Republic; 4 Faculty of Medicine in Pilsen, Charles University, Prague, Czech Republic; 5 Institute of Molecular Genetics of the Czech Academy of Sciences, Prague, Czech Republic

**Keywords:** Proteomics, Heart failure, Experimental models of disease, Translational research, Cardiac hypertrophy

## Abstract

Mechanisms of right ventricular (RV) dysfunction in heart failure (HF) are poorly understood. RV response to volume overload (VO), a common contributing factor to HF, is rarely studied. The goal was to identify interventricular differences in response to chronic VO. Rats underwent aorto-caval fistula (ACF)/sham operation to induce VO. After 24 weeks, RV and left ventricular (LV) functions, gene expression and proteomics were studied. ACF led to biventricular dilatation, systolic dysfunction and hypertrophy affecting relatively more RV. Increased RV afterload contributed to larger RV stroke work increment compared to LV. Both ACF ventricles displayed upregulation of genes of myocardial stress and metabolism. Most proteins reacted to VO in a similar direction in both ventricles, yet the expression changes were more pronounced in RV (p_slope_: < 0.001). The most upregulated were extracellular matrix (POSTN, NRAP, TGM2, CKAP4), cell adhesion (NCAM, NRAP, XIRP2) and cytoskeletal proteins (FHL1, CSRP3) and enzymes of carbohydrate (PKM) or norepinephrine (MAOA) metabolism. Downregulated were MYH6 and FAO enzymes. Therefore, when exposed to identical VO, both ventricles display similar upregulation of stress and metabolic markers. Relatively larger response of ACF RV compared to the LV may be caused by concomitant pulmonary hypertension. No evidence supports RV chamber-specific regulation of protein expression in response to VO.

## Introduction

Due to current scope of heart failure (HF) epidemics, exploration of new approaches to prevent or stabilize HF is a priority for research. One of important milestones of HF progression is the onset of right ventricular failure due to transition of initially left ventricular disease into biventricular HF with dismal prognosis^1^. Until recently, right ventricle (RV) received little attention and the mechanisms responsible for RV dysfunction are poorly understood^2,3^. RV differs in many aspects from the LV, having different embryonic origin, geometry, wall thickness and operating pressures^2–6^. Whether these differences translate into a “RV-specific” response to increased hemodynamic stress is unknown. Such interventricular differences may theoretically represent a target for chamber-specific therapies.

RV dysfunction can develop due to pressure overload resulting from pulmonary hypertension and this scenario is often investigated^3,7^. However, the response of RV to chronic volume overload (VO), a common contributing factor to HF, is studied much less^8^. The most frequent cause of VO of RV is severe tricuspid regurgitation that accompanies all forms of advanced HF^9^. Chronic VO of RV also occurs in patients with congenital heart disease^10^ or after implantation of left-ventricular assist device^11,12^. Therefore, chronic VO of RV is common, yet understudied condition.

Few studies examined molecular mechanisms of volume-induced RV dysfunction^8,10,13–17^. It is unclear whether RV response fundamentally differs from LV response to excessive VO. Function of ventricles exposed to VO is influenced by altered loading and geometry, so only load-independent assessment by simultaneous invasive pressure–volume analysis can provide insight into chamber-specific responses to VO. Advantageous model to study interventricular differences in stress response is chronic infrarenal aorto-caval fistula (ACF), a condition that imposes identical VO both on the left and the right heart, leading to biventricular cardiac hypertrophy, dilatation, dysfunction and symptomatic heart failure^18,19^.

The goal of this study was to characterize the functional and molecular response of RV and LV to chronic VO and to identify hemodynamic factors that drive VO chamber remodeling using pressure–volume analysis, echocardiography, quantitative proteomics, and gene expression of selected genes previously implicated in development of RV failure^14^.

## Results

### Cardiac structure and function in response to VO

After 24 weeks of VO by ACF, we observed massive biventricular hypertrophy, relatively more pronounced on the right side (RV: + 150%, LV: + 70%, both *p* < 0.0001), increased atrial weight and increased lung weight due to congestion (Table 1). 70% of ACF animals showed clinical HF signs. ACF rats had similar tibial length as controls but were heavier due to presence of congestion.

**Table 1:** Baseline characteristics and echocardiography.

	Control	ACF	*P* (t-test)	Fold-change ACF vs control
Body weight (BW), g	550 ± 50	610 ± 70	0.0035	1.1
Tibial length, mm	43 ± 1	44 ± 1	0.3668	1.0
Heart weight/BW, g kg^−1^	3.0 ± 0.2	5.9 ± 0.8	< 0.0001	2.0
LV weight/BW, g kg^−1^	2.0 ± 0.2	3.4 ± 0.5	< 0.0001	1.7
RV weight/BW, g kg^−1^	0.53 ± 0.05	1.3 ± 0.2	< 0.0001	2.5
Atrial weight/BW, g kg^−1^	0.33 ± 0.06	1.0 ± 0.2	< 0.0001	3.0
Lung weight/BW, g kg^−1^	3.5 ± 0.4	5 ± 1	< 0.0001	1.4
Heart failure score (0–7)	0.03 ± 0.11	1.6 ± 1.5	< 0.0001	53
**Echocardiography: left ventricle (LV)**				
LV end-diastolic dimension, mm	6.7 ± 0.6	12 ± 1	< 0.0001	1.8
LV posterior wall thickness, mm	2.6 ± 0.3	2.2 ± 0.2	< 0.0001	0.8
Relative wall thickness	0.8 ± 0.1	0.33 ± 0.05	< 0.0001	0.4
LV fractional shortening, %	58 ± 5	38 ± 6	< 0.0001	0.7
Heart rate, min^−1^	460 ± 30	370 ± 40	< 0.0001	0.8
Stroke volume, ml	0.29 ± 0.08	1.5 ± 0.3	< 0.0001	5.2
Cardiac output, ml min^−1^	130 ± 30	500 ± 100	< 0.0001	3.8
Mitral regurgitation grade (1–4)	0.3 ± 0.5	1.3 ± 0.9	0.0004	4.3
**Echocardiography: right ventricle (RV)**				
RVD1, mm	3.6 ± 0.3	7 ± 1	< 0.0001	1.9
RVD2, mm	3.5 ± 0.3	7 ± 1	< 0.0001	2.0
RVD3, mm	9.7 ± 0.7	14 ± 1	< 0.0001	1.4
RV diastolic area, mm^2^	32 ± 2	90 ± 20	< 0.0001	2.8
RV FAC, %	49 ± 4	40 ± 10	0.0037	0.8
TAPSE, mm	3.1 ± 0.2	3.9 ± 0.7	< 0.0001	1.3
RV global strain, %	−9 ± 2	−13 ± 3	0.0005	1.4
RV global strain rate, s^−1^	1.4 ± 0.3	2.0 ± 0.4	< 0.0001	1.4
Tricuspid regurgitation grade (1–4)	0.4 ± 0.5	1.3 ± 0.8	0.0012	3.3

Values are expressed as mean ± SD.

BW, body weight; LV, left ventricle; RV, right ventricle; RVD, right ventricular diameter; FAC, fractional area change; TAPSE, tricuspid annular plane systolic excursion.

**Figure 1:**
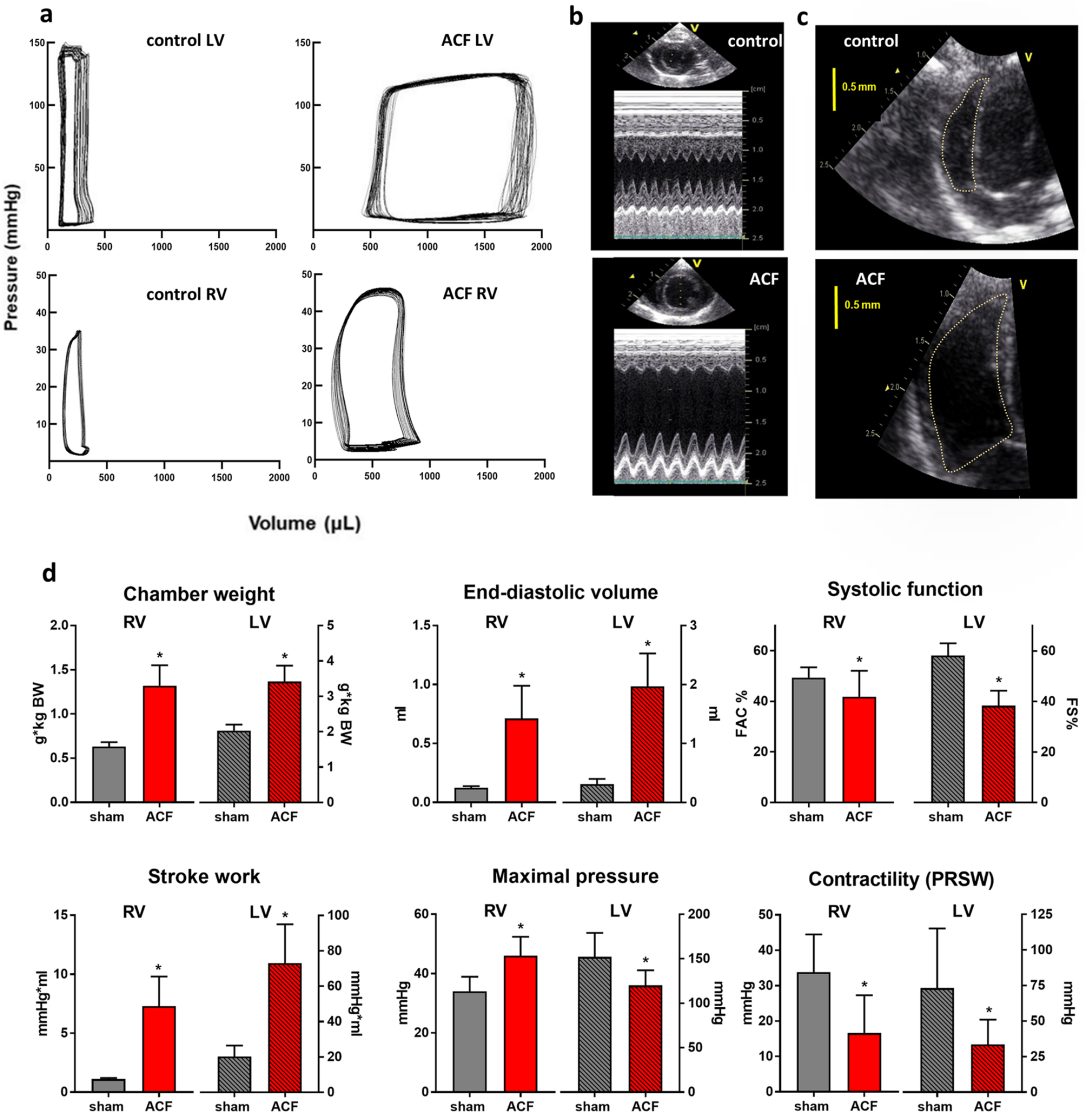
Volume overload-induced cardiac remodeling of right (RV) and left (LV) ventricle. (**a**) Representative example of pressure–volume (PV) loops in HF and controls. (**b**) Echocardiographic parasternal short axis view of LV in M-mode, (**c**) 4-chamber view of RV in controls and ACF. (**d**) Hemodynamic parameters and PV analysis results in ACF (N = 26) and controls (N = 16). Data are presented as means ± SD. *p < 0.05 vs controls.

Echocardiography (Table 1, Fig. 1a–c) confirmed LV chamber dilatation, wall thinning, eccentric remodeling, relative wall thinning and depressed global LV systolic function (LV fractional shortening: by − 30%) in ACF animals. Due to the systemic shunt, cardiac output was increased in ACF. Right ventricle showed enlargement and reduced global systolic function (RV FAC: by − 20%). Regional echocardiographic RV functional parameters (tricuspid annular plane systolic excursion—TAPSE, RV systolic strain) were influenced by marked ventricular remodeling/dilatation and overestimated RV systolic function.

Invasive hemodynamics and pressure–volume analysis (Table 2, Fig. 1d) showed reduced systemic mean and diastolic blood pressure in ACF. End-diastolic filling pressures were increased in both ventricles, but more in the left ventricle. End-diastolic (and end-systolic) volumes were similarly increased in both ventricles. Maximal LV pressure and LV dP/dt_max_ were reduced, but maximal RV pressure and RV dP/dt_max_ were increased in ACF compared to controls. The difference between maximal RV pressure and LV end-diastolic pressure that reflects transpulmonary pressure gradient (and precapillary component of pulmonary hypertension), was increased in ACF by 25% compared to controls. Ventricular stroke work was markedly increased in both ventricles, but the increase was relatively larger in the right ventricle (RV: 6.4-fold and LV: 3.5-fold, respectively). Load-independent measure of chamber contractility—preload-recruitable stroke work (PRSW), was indeed significantly reduced both in ACF RV (by − 30%, *p* = 0.0154) and LV (by − 60%, *p* = 0.0021), confirming depressed systolic chamber function. LV diastolic function was impaired (relaxation constant tau increased by 50%, *p* < 0.0001; relaxation rate dP/dt_min_ decreased twofold, *p* < 0.0001) but RV diastolic function was not influenced by ACF.

**Table 2:** Hemodynamic data from pressure–volume analysis.

	Control	ACF	*P* (t-test)	Fold-change ACF vs control
**Systemic circulation**				
SBP, mmHg	140 ± 30	130 ± 20	0.1164	0.9
DBP, mmHg	110 ± 30	80 ± 10	< 0.0001	0.7
MBP, mmHg	130 ± 30	110 ± 20	0.0024	0.8
PP, mmHg	29 ± 6	47 ± 8	< 0.0001	1.6
SVR, mmHg.min.ml^−1^	1 ± 0.3	0.21 ± 0.05	< 0.0001	0.2
**Left Ventricle (LV)**				
LV EDP, mmHg	6 ± 2	12 ± 4	< 0.0001	2.0
LV EDV, ml	0.31 ± 0.09	2.0 ± 0.5	< 0.0001	6.5
LV mass/EDV, g.ml^−1^	3.8 ± 0.9	1.1 ± 0.2	< 0.0001	0.3
LV ESV, ml	0.02 ± 0.01	0.5 ± 0.3	< 0.0001	25
LV max pressure, mmHg	150 ± 30	120 ± 20	< 0.0001	0.8
LV max wall stress, mmHg.ml.g^−1^	40 ± 10	120 ± 30	< 0.0001	3.0
Stroke work, mmHg.ml	20 ± 6	70 ± 20	< 0.0001	3.5
dP/dt_max_, mmHg.s^−1^	10,000 ± 4000	9000 ± 3000	0.1504	0.9
PRSW, mmHg	70 ± 40	30 ± 20	0.0021	0.4
dP/dt_min_, mmHg.s^−1^	−11,000 ± 3,000	−5000 ± 2000	< 0.0001	0.5
Tau, ms	11 ± 2	17 ± 4	< 0.0001	1.5
**Right ventricle (RV)**				
RV EDP, mmHg	4 ± 1	6 ± 2	0.0028	1.5
RV EDV, ml	0.12 ± 0.01	0.7 ± 0.3	< 0.0001	5.8
RV mass/EDV, g.ml^−1^	2.4 ± 0.3	1.2 ± 0.3	< 0.0001	0.5
RV ESV, ml	0.038 ± 0.005	0.3 ± 0.2	< 0.0001	7.9
RV max pressure, mmHg	34 ± 5	46 ± 6	< 0.0001	1.4
RV max pressure—LVEDP gradient, mmHg	28 ± 6	35 ± 5	0.0023	1.3
RV max wall stress, mmHg.ml.g^−1^	15 ± 3	36 ± 9	< 0.0001	2.4
Stroke work, mmHg.ml	1.1 ± 0.1	7 ± 3	< 0.0001	6.4
dP/dt_max_, mmHg.s^−1^	2200 ± 800	2900 ± 800	0.0265	1.3
PRSW, mmHg	30 ± 10	20 ± 10	0.0154	0.7
dP/dt_min_, mmHg.s^−1^	−1600 ± 300	−1600 ± 400	0.9405	1.0
Tau, ms	20 ± 10	20 ± 10	0.9954	1.0

Values are expressed as mean ± SD.

SBP, systolic blood pressure; DBP, diastolic blood pressure; MBP, mean blood pressure; PP, pulse pressure; SCR, systemic vascular resistance; LV, left ventricle; RV, right ventricle; PRSW, preload-recruitable stroke work; EDP, enddiastolic pressure; EDV, enddiastolic volume; ESV, endsystolic volume. For other abbreviations, see text.

The RV and LV exposed to volume overload (VO) could possibly differ in action potential duration or trabecular contraction force, therefore we measured these parameters and presented the results in Fig. 3a,b. Parameters do not significantly differ between the ventricles. ACF had profound effect on duration of action potential (APD_90_) that was similarly prolonged in both VO-exposed ventricles compared to controls.

### Expression of target genes in response to VO

**Figure 2:**
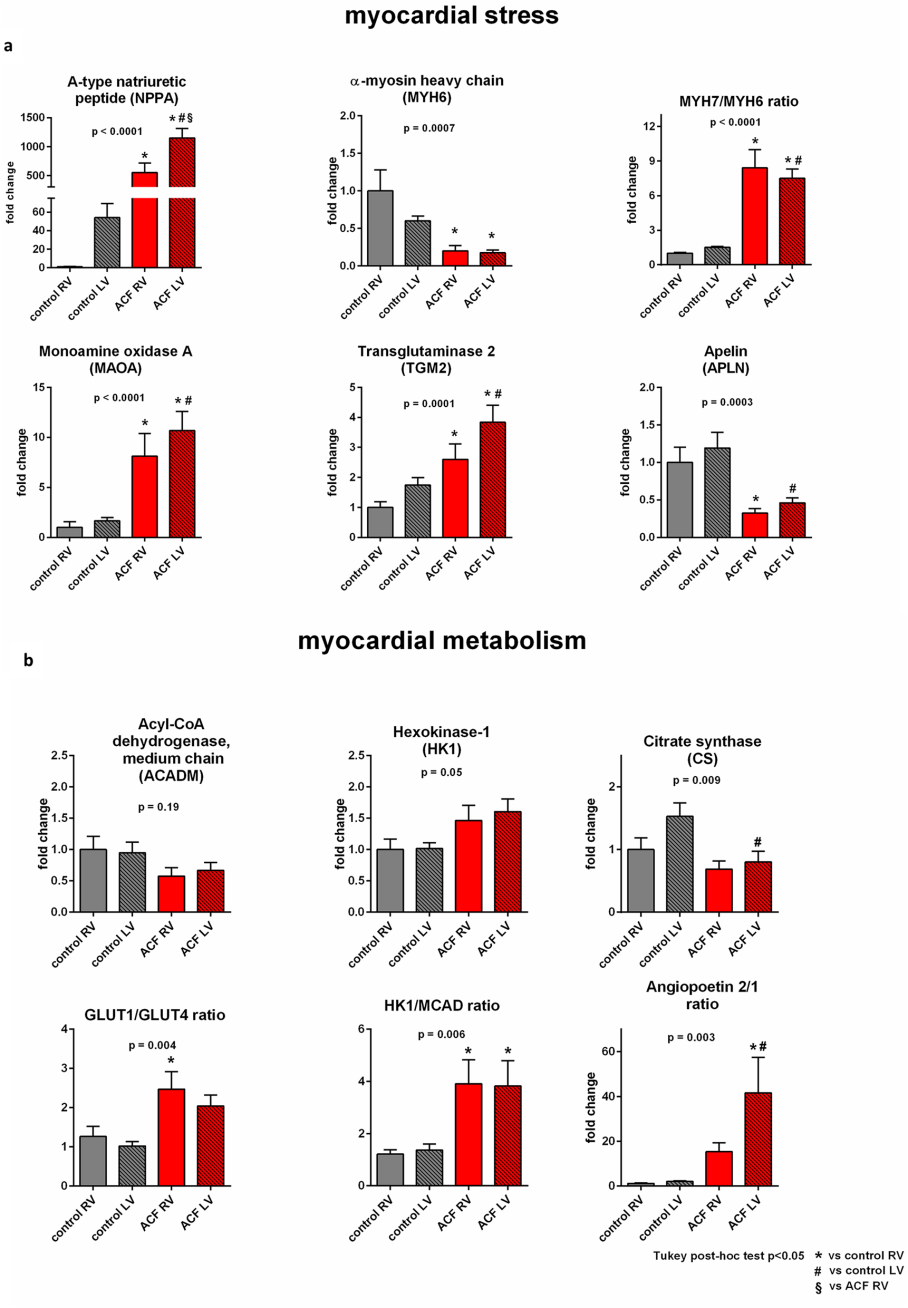
Gene mRNA expression analysis of cardiac markers of stress and metabolism. Gene mRNA expression analysis of selected genes that reflect (**a**) myocardial stress, (**b**) substrate metabolism and bioenergetics. Data are presented as means ± SEM. Changes normalized to control RV. N = 12 in each group. *p* value: ANOVA, and Tukey post-hoc tests.

Gene mRNA expression (qPCR) of selected genes (for list, see methods supplement) showed that ACF led to upregulation of myocardial stress genes (Fig. 2a): natriuretic peptide A (*Nppa*, more upregulated in LV than in RV) and increased myosin heavy chain isotype ratios (*Myh7/6*), mostly due to downregulated *Myh6* gene. Monoamine oxidase-A *(Maoa)* and transglutaminase-2 (*Tgm2*), genes previously associated with ACF^20^, were similarly upregulated in RV and LV, apelin^7^ (*Apln*) was similarly downregulated in both ACF ventricles, with no effect of VO on apelin receptor *(Aplnr)* (Figure S1).

Metabolic genes showed a pattern consistent with HF-induced reprogramming (Fig. 2b). Increased ratio of gene expression changes of Hexokinase 1 (*Hk1*) to Medium chain Acyl CoA dehydrogenase (*Acadm*) reflected enhanced glycolysis over the fatty acid oxidation in both ventricles. Increased ratio of glucose transporters *Glut1/Glut4* was consistent with increased non-insulin dependent glucose uptake in ACF ventricles, more pronounced in RV. In both ventricles, VO had no effect on genes related to tissue hypoxia and angiogenesis (*Vegf, Hif-1a*), only Angiopoetin 2/Angiopoetin 1 ratio was significantly increased in both ACF ventricles, more in the LV. Expression of genes of cGMP-dependent signaling pathway (natriuretic peptide receptor 1 and 2*: Npr1,2,* soluble guanylate cyclase: *Gucy1a3,* cGMP-dependent protein kinase*: Pkg,* phosphodiesterase 5: *Pde5a,* Phosphodiesterase 9: *Pde9a*), was not consistently affected by VO (Figure S1). Myocardial cGMP concentration was increased in ACF RV and LV (Figure S2), likely due to elevated levels of natriuretic peptides.

**Figure 3:**
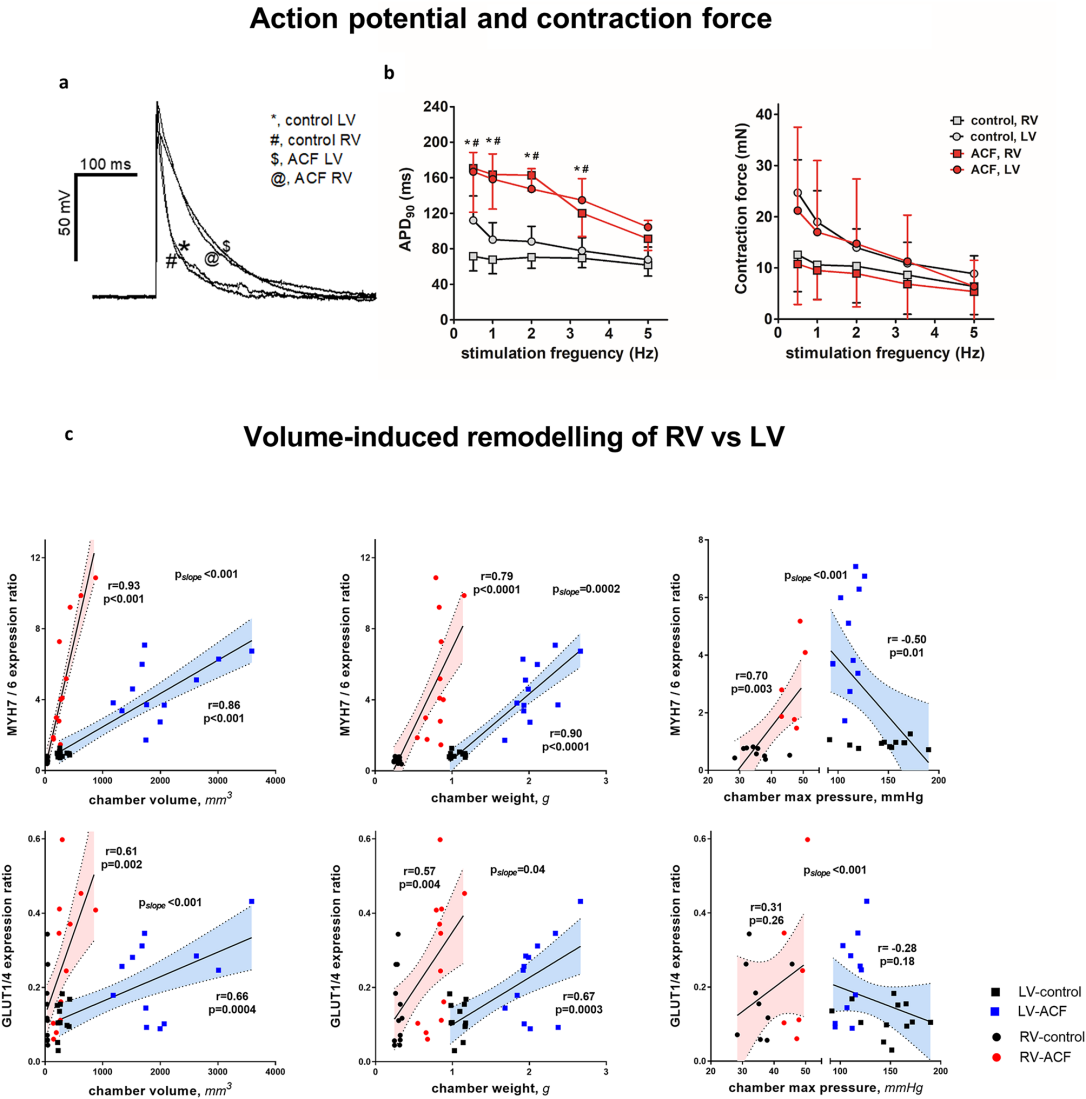
Biventricular differences in contraction force, action potential duration and mRNA expression regulation in volume overload from aorto-caval fistula (ACF). (**a**) Example of action potential recording from isolated papillary muscle. (**b left**) Action potential duration at 90% repolarization (APD90) in RV and LV papillary muscles in controls and in ACF rats. Measurements at stimulation frequencies of 0.5, 1, 2, 3.3 and 5 Hz. *, *p* < 0.05, RV control vs. RV ACF; #, *p* < 0.05, LV control vs. LV ACF (3-way ANOVA). (**b right**) Maximal contraction force in RV and LV papillary muscles in control and ACF rats. (**c**) Relation of hypertrophy determinants to gene mRNA expression patterns in RV and LV. Note discordant relations between gene expression and ventricular pressures; concordant but steeper relation of RV compared to LV. r: Pearson´s correlation coefficient, p: *p*-value of correlation. The line represents linear regression with 95% confidence bands. p_slope_ denotes the difference between regression slopes.

To identify chamber-specific responses to VO, we correlated individual hemodynamic parameters with sensitive gene expression markers of myocardial stress response (*Myh7/6 ratio*)^13,15^ and myocardial substrate metabolism (*Glut1/4 ratio*)^21^ (Fig. 3c, Table 3). The variable with strongest correlation to stress and/or metabolic response in both ventricles was end-diastolic volume and ventricular mass. For the same level of end-diastolic volume or mass increase, the gene expression in the RV was more pronounced (i.e. steeper regression slope) than in the LV. Such pattern, albeit weaker, was observed with other target genes. Directionality of gene expression-hemodynamics relations in RV corresponded to LV, with an important exemption of peak ventricular pressure (Fig. 3c). In the RV, higher maximal pressure linked with more pronounced gene expression, while in the LV, lower maximal pressure was associated with more remodeling. This indicates that ACF-induced changes in the right ventricle combine VO with simultaneous pressure overload, and lead to more pronounced gene expression changes, while the left ventricle is in fact “pressure-unloaded” due to presence of ACF combined with lower systemic pressures.

**Table 3:**
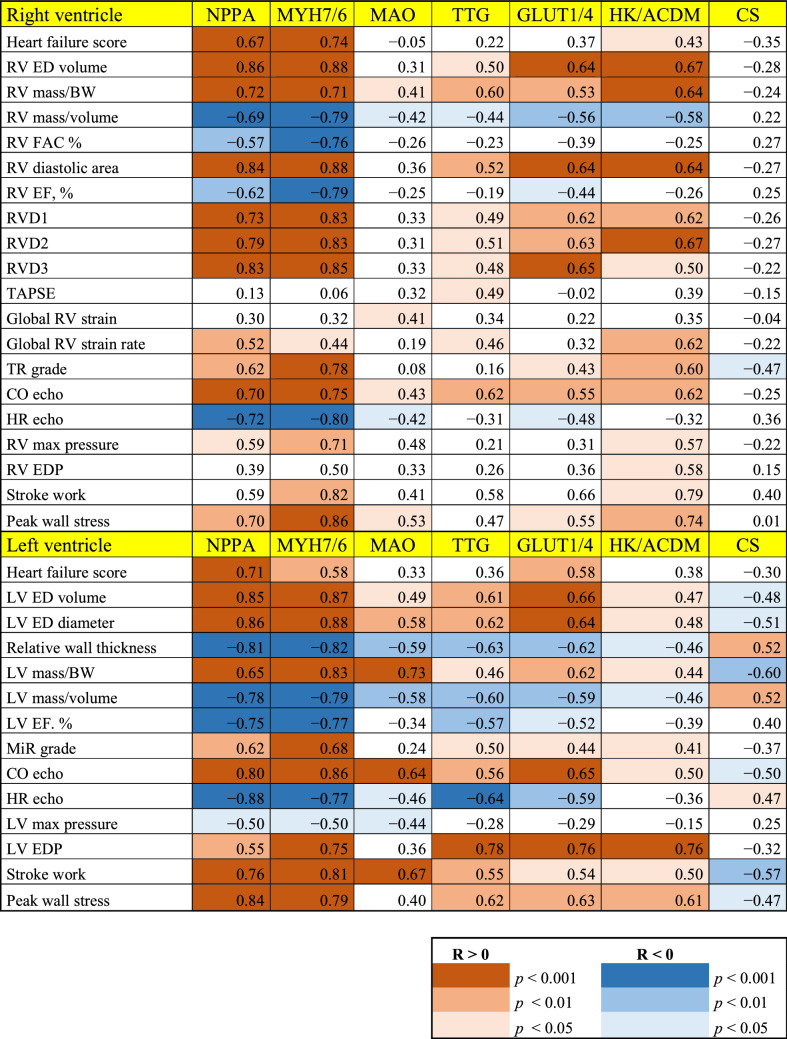
Correlation matrix of cardiac function parameters and gene expression in aggregated dataset.

Values are Pearson’s r. *p* values are coded by colors.

RV, right ventricle; ED, end-diastolic; BW, body weight; FAC%, fractional area change; RVD, right ventricular diameter; TR, tricuspid regurgitation grade; CO, cardiac output; EDP, enddiastolic pressure; MiR, mitral regurgitation; LV, left ventricle; EF, ejection fraction. For other abbreviations: see text.

### Ventricular proteome response to VO

**Figure 4:**
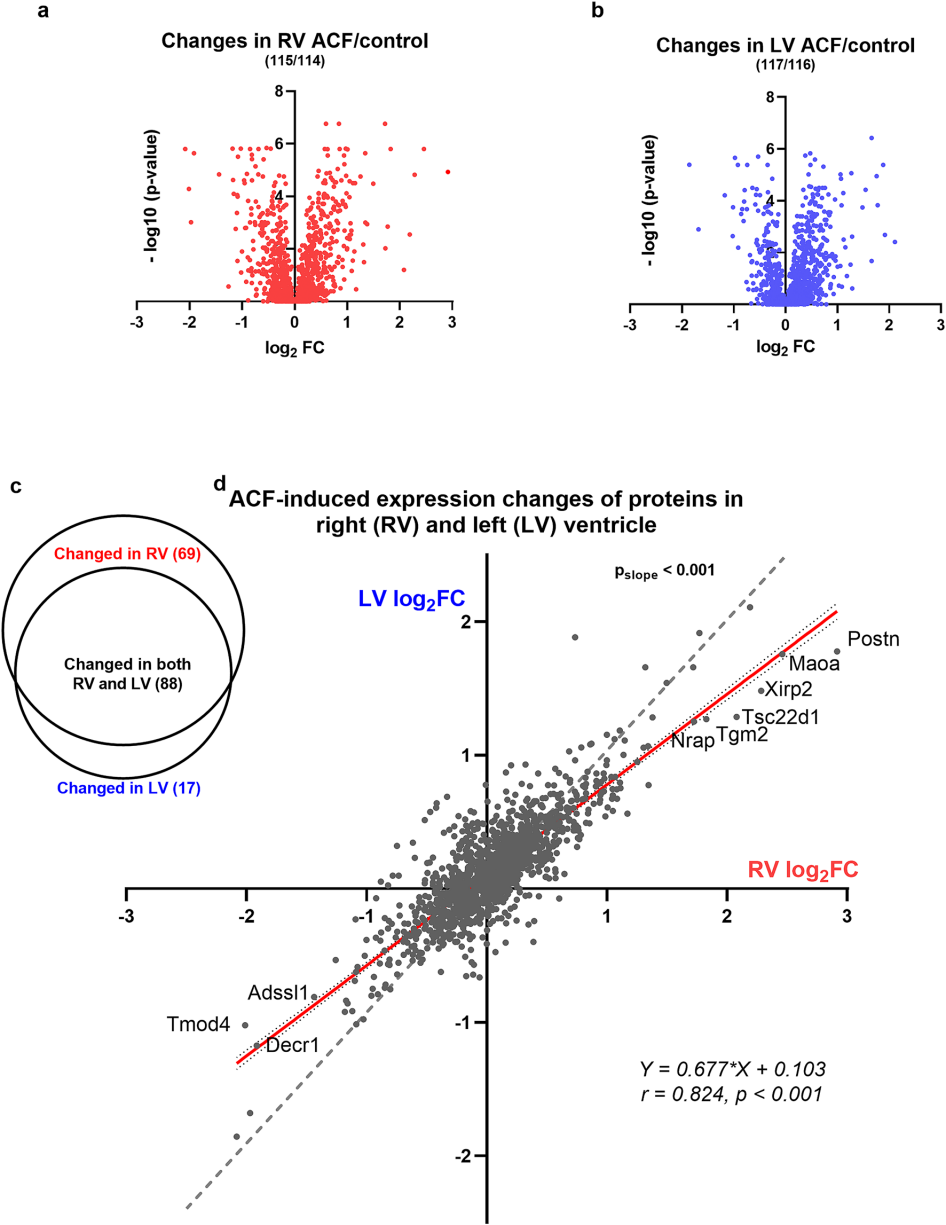
iTRAQ proteomic analysis of biventricular response to volume overload due to ACF. (**a**, **b**) Vulcan plot of identified proteins in RV and LV. X axis represents log2 of fold change compared to controls, Y axis -log10 of *p*-value. (**c**) Venn diagrams of differentially regulated proteins in ACF compared to controls (significant difference with threshold > 1.5-fold). (**d**) ACF-induced changes (log2 of fold-) in RV (X axis) vs LV (Y axis). Note departure of the regression line (with 95% CI, red) from identity line (dashed), towards RV. The proteins with relatively highest differential regulation in RV are labeled in red. For abbreviations, see text.

**Figure 5:**
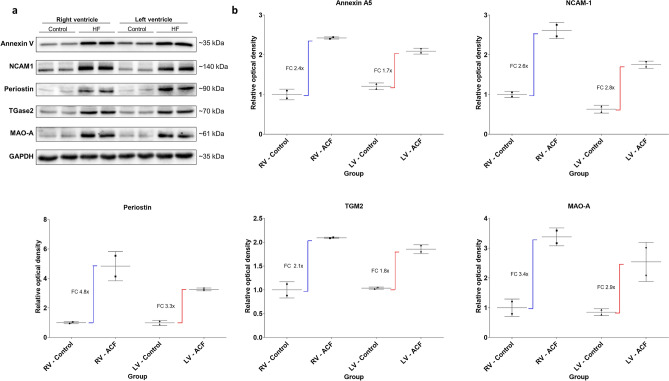
Confirmation of the differential expression of selected proteins using western blot analysis with specific antibodies and densitometry. (**a**) Western blot analysis using specific antibodies. Pooled myocardial samples (40 μg) were loaded and separated in duplicates. GAPDH was used as a loading control. NCAM-1 (Neural cell adhesion molecule 1), TGM2 (Tissue-type transglutaminase), MAO-A (Monoamine oxidase type A). (**b**) Quantitative densitometry of the blots confirms more pronounced upregulation of the proteins in RV compared to LV as observed in the proteomic analysis.

Triplicate iTRAQ-based LC–MS/MS proteomic analysis of pooled samples of RVs and LVs of ACF and control animals identified over 3000 cardiac proteins. 1487 proteins were detected in all three replicates and provided quantitative expression data with adjusted p-value calculations. Only the proteins identified with at least 2 unique peptides (1372 proteins in total) were further considered (full list of identified proteins is provided as a supplementary dataset). 19 proteins were differentially expressed in both ACF ventricles (≥ twofold change up or down with adjusted *p*-value < 0.05 vs. controls), 25 additional proteins met the same differential expression criteria only in the RV (all also trending in LV) and 3 proteins were differentially expressed in the LV only (also trending in RV) (Table 4, Fig. 4). The differentially expressed proteins due to ACF were thus highly concordant between RV and LV without evidence of any protein expression changes specific for either ventricle. Yet, the magnitude of the ACF-induced expression changes was more pronounced in the RV (approximate ratio across all quantified proteins 1:0.67, Fig. 4) than in the LV (p_slope_: < 0.001). The individual list of the most differentially regulated proteins (> twofold change up or down compared to controls) is presented in Table 4 and is discussed below. Expression changes of selected proteins were confirmed by western blotting using specific antibodies and densitometry (Fig. 5).

**Table 4:**
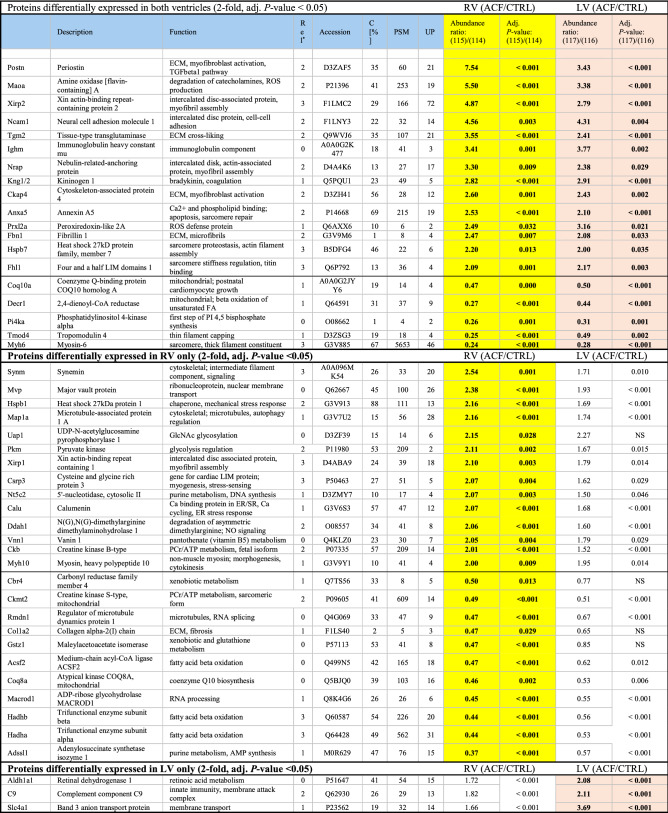
Differentially regulated proteins in RV vs LV due to volume overload.

Rel*, relevance to known association with myocardium or hearth failure (HF); 0, no reports; 1, known to be in the heart; 2, associates with HF phenotype; potentially modifies HF; 3, causally linked to HF; mutations are causal to HF/cardiomyopathy; C [%], percentual coverage; UP, number of unique peptides; PSM, number of post translational modification.

## Discussion

The study describes biventricular changes in myocardial function and protein composition induced by chronic VO due to ACF. In response to ACF, both ventricles displayed eccentric hypertrophy, reduced contractility, prolonged duration of action potential, upregulation of genes associated with myocardial stress (*Nppa, Myh7/6 ratio*) and expression changes in substrate metabolic genes consistent with enhanced glycolysis and reduced fatty acid oxidation. By proteomic analysis, we identified several novel cardiac proteins differentially regulated by ACF with qualitatively concordant changes in both ventricles. The principal finding is that in response to identical surplus volume load, the pattern of proteome alterations is almost identical between ventricles, but the magnitude of changes is relatively more pronounced in the RV than in the LV. More pronounced changes on the right side can be explained by relatively larger increase in stroke work by RV compared to LV. Therefore, quantitative differences in protein expression between ventricles are explainable by hemodynamics, rather than by an existence of a “chamber-specific” regulation^10^. Study suggests that one possible way how to preserve RV function and to prevent adverse RV remodeling in HF could be to lower excessive hemodynamic loading.

### VO-induced changes in biventricular hemodynamics, structure and function

Echocardiography showed eccentric remodeling, relative wall thinning, increased wall stress and depressed volumetric indices of function of both ventricles exposed to ACF. Indices of regional RV function (TAPSE, RV strain and strain rate) were paradoxically increased in ACF, likely due to confounding influence of chamber geometry, grossly changed by ACF. Therefore, TAPSE or strain deformation analysis overestimate RV contractility in volume overloaded ventricles. Limited utility of regional RV function indexes is also supported by low correlation of TAPSE, RV global strain and strain rate with gene expression of markers of myocardial remodeling (Table 3), in contrast to volumetric parameters.

Hemodynamic results are consistent with previous reports, although no study utilized pressure–volume analysis of both ventricles simultaneously in this model. While LV dP/dt_max_ showed a trend toward reduction in ACF compared to control^19,22^, RV dP/dt_max_ was increased in ACF^13,19^, reflecting either afterload-dependence of this parameter or heterometric (Frank-Starling) adaptation to increased load. Load-independent measure of chamber contractility—preload-recruitable stroke work (PRSW) was reduced in both ventricles, confirming indeed depressed systolic function^15^. Diastolic function (dP/dt_min_ and tau) was impaired in ACF LV, which might contribute to the development of pulmonary hypertension^23^.

Despite both ventricles handle the same increase in cardiac output in ACF, the increase in myocardial mass was relatively larger in the RV compared to LV (2.5 vs 1.7-fold, corresponding to LV/RV ratio of 0.7). Larger impact of ACF on RV compared to LV was noticed previously^16,19,24^. The explanation could be in a different stress response compensation, or due to difference in regulation of cardiac growth between ventricles^25^, or it can be explained hemodynamically. Our data support the latter mechanism. Pressure–volume data showed that ACF RV has to bear relatively higher increment of hemodynamic burden than LV. RV stroke work is increased 6.4-fold while LV stroke work is increased 3.5-fold in ACF compared to normal. Larger loading of the RV can be explained by pulmonary hypertension that adds to VO of the right heart, likely due to latent pulmonary vascular disease^26^ that is reflected by increased surrogate of transpulmonary pressure gradient (due to lack of direct PA pressure measurement, estimated here as RV peak pressure-LVEDP). Pulmonary vascular disease in ACF develops due to chronic elevation of pulmonary venous pressure^23^ and due to excessive pulmonary blood flow^16,24,26^.

The response to volume overload (VO) could possibly change action potential duration or trabecular contraction force in RV or LV. Isolated ventricular trabeculae showed no impact of ACF in developed force or force-frequency relationship supporting minimal interventricular differences in response to VO. Some^22,27^ but not all^28,29^ previous studies demonstrated reduced contractility of isolated cardiomyocytes^22^ or isolated papillary muscle preparations^27,29^ from rats with VO due to ACF. Further and more detailed analyses of RV and LV myofilament sensitivity are mandated. Both RV and LV from ACF group showed profound electrophysiological remodeling with almost doubling of action potential duration (APD) compared to controls. Prolongation of APD may be a compensatory mechanism how to maintain contraction strength in VO. APD prolongation is pro-arrhythmogenic and together with other mechanisms can contribute to increased risk of arrhythmic sudden death in volume-overloaded hearts^30^.

### VO-induced changes in biventricular mRNA gene expression

VO led to upregulation of gene for natriuretic peptide A (*Nppa*) in both ventricles^8,13,31^. ACF-induced upregulation of ANP mRNA was massive and it was more pronounced in LV than in RV. In most HF animal models, including ACF^32^, as well as in humans with cardiac overload, the progression of cardiac hypertrophy into HF is associated with reduced expression of *Myh6* gene, coding α-myosin heavy chain, either absolutely or in relation to *Myh7,* gene of β-myosin heavy chain^13,32,33^. Change of *Nppa* gene expression and *Myh7/6* ratio are therefore the most consistent molecular markers of HF and were upregulated in both VO-exposed ventricles. At a given surplus of mass, expression of *Nppa* or *Myh7/6* were more pronounced in RV than in LV (Fig. 3c). Observed changes in *Myh7/6* mRNA expression were consistent with proteomic analysis. In agreement with mRNA data (Fig. 2), ACF led to higher increase in protein MYH7/6 ratio in RV (5.2) than in LV (4.07). MYH6 protein expression was markedly significantly downregulated in both ACF ventricles (RV by 0.24, LV by 0.28), while MYH7 was mildly significantly upregulated in RV (by 1.25) and mildly non-significantly upregulated in LV (by 1.14) (Supplementary proteomic dataset). Correlation analysis (Table 3) also showed that changes of *Myh7/6* and *Nppa* expression changes are linked with similar hemodynamic variables, and are likely co-regulated, in contrast to metabolic genes.

Volume overload led to change in genes of myocardial substrate metabolism and bioenergetics, such as increased *Glut1/4* ratio, indicative of enhanced insulin-independent glucose uptake, and increased *Hk1/Mcad* ratio, indicative of enhanced glycolysis with reduced transcription of genes of fatty acid β-oxidation (*Mcad*). These changes were demonstrated in both ventricles. Similar pattern of metabolic gene transcription program was observed previously in pressure-overloaded ventricles, including RV^21^. In summary, metabolic response to stress resembles a reactivation of fetal gene expression program and is uniform in terms of chamber (RV vs LV) or overload etiology^21^.

We did not find altered expression of genes coding *Vegfa* and *Hif1a* in failing ventricles, but we observed increased ratio of *Angpt2/Angpt1* mRNA (coding antiangiogenic angiopoietin-1 and angiopoietin-2), mostly in ACF LV, indicative of altered angiogenic signaling, similar to the response to myocardial infarction^34^. There were no consistent differences in genes of cGMP-dependent signaling pathway, speaking against relevance of this pathway in response to VO. Yet myocardial cGMP concentration was increased in ACF ventricles, probably reflecting stimulation of NP receptor-associated (particulate) guanylate cyclase by elevated natriuretic peptides.

VO in both ventricles led to downregulation of apelin, a small peptide with cardioprotective, inotropic and angiogenic properties that has contra-regulatory effects to renin-angiotensin system and acts via apelin receptor (*Aplnr*)^35^. Downregulation of myocardial apelin was previously described in failing pressure-overloaded LV or RV^7,35^, but this is the first study that links apelin to volume overload–induced remodeling.

### VO-induced changes in biventricular proteome

VO-induced changes in protein abundance were mostly similar between RV and LV. All differentially expressed proteins were either concordantly altered (≥ 2-fold change) in both ventricles, or significantly altered only in one ventricle with concordant and/or not significant change of expression in the second ventricle. The data thus provide no support for chamber-specific protein expression patterns in response to similar hemodynamic stress, as proposed previously on basis of interventricular differences in physiology and embryonic origin^10^. Yet, protein deregulation was more pronounced in the right ventricle than in left with ratio of 1:0.67, i.e. regression line was tilted from the line of equivalence towards RV (Fig. 4d). Interestingly, this ratio is numerically close to the ratio of ventricular mass increments (RV/LV mass ratio 1:0.7). Proteome data also agree with hemodynamic data, as ACF led to more pronounced change in stroke work and myocardial mass in RV than in LV.

Most proteins emerged as differentially and correspondingly regulated in both ACF ventricles, compared to sham-operated ventricles, some of them for the first time associated with response to VO. The analysis of these proteins helps to understand VO-induced myocardial remodeling shared by both ventricles and they will be discussed by functional groups.

#### Proteins related to extracellular matrix (ECM)

The most upregulated protein in ACF ventricles was **periostin** (POSTN)—a non-structural component of ECM, marker of myofibroblasts, cells necessary for cardiac adaptive healing and fibrosis^36^. Periostin assists in deposition of fibronectin-rich ECM and collagen crosslinking^37^, cardiomyocyte dedifferentiation^38^ and is extensively upregulated by TGFβ1, angiotensin II, infarction or hemodynamic overload, including VO^39,40^. VO-driven upregulation is almost twice in RV than in LV. Another TGFβ1-regulated protein upregulated in ACF hearts is c**ytoskeleton associated protein 4** (CKAP4), known to positively correlate with activated myofibroblast markers in both mouse and human cardiac tissue and to be negative modulator of fibroblast activation in injured heart^41^. We report again strong upregulation of **tissue-type transglutaminase 2** (TGM2) in both ACF ventricles^20^, both on protein or mRNA level. TGM2 is responsible for crosslinking and stiffening of ECM and it was implicated in development of HF^42^. Another upregulated ECM protein is **fibrillin 1,** a constituent of ECM microfibrils that is enhanced in ANGII-induced cardiac fibrosis^43^.

#### Sarcomeric, cytoskeletal, and cell–cell interaction proteins

Second large group of upregulated proteins in ACF were cytoskeletal proteins, sarcomeric proteins and proteins responsible for cell–cell interaction/force transduction. We report here an upregulation of two Xin actin-binding repeat-containing proteins **XIRP1** and **XIRP2**. These proteins with almost cardiac-specific expression are associated with intercalated disks and play a role in myofibril assembly and repair^44^. XIRP2 modulates the effects of ANG-II on cardiac hypertrophy, fibrosis and myosin isotype switch^45^, regulates voltage-gated K changes (KV1.5)^46^, and was found to be upregulated in RV by experimental volume-overload^47^. XIRPs may therefore represent potential markers of cardiac injury. Mutations of both XIRPs were associated with arrhythmic sudden cardiac death and prolonged action potential^46^, a feature present also in VO-ACF hearts (Fig. 3b).

From other sarcomeric proteins, we found downregulated gene for **α-myosin heavy chain** (**MYH6)**, consistently with our targeted mRNA analysis and previous studies^33^. Across species and types of overload, the downregulation of MYH6 gene is one of the hallmarks of HF^48^. ACF ventricles displayed also two-fold upregulation of non-muscle **myosin 10** and downregulation of **tropomodulin 4**. Upregulated cytoskeletal signaling protein **four and-a-half LIM domains 1 (FHL-1**) which binds to and regulates titin stiffness was already linked to VO-induced cardiac LV remodeling in rats^31^. Another sarcomeric stress-sensing element upregulated in ACF is cardiac **cysteine and glycine-rich protein 3 (CSRP3 aka MLP)**^49^. Mutations in FHL1^50^ and CSRP3 are known to cause cardiomyopathies. VO-ventricles showed an upregulation of **annexin 5**, intracellular protein that participates in Ca^2+^ handling, apoptosis and sarcolemma repair and is upregulated in failing human myocardium^51^.

One of the most upregulated proteins in ACF ventricles was **neural cell adhesion molecule 1** (NCAM1, 4.5-fold**),** a plasma membrane protein relevant for cardiomyocyte cell–cell interactions. NCAM1 is, similarly to identified ECM proteins, regulated by TGFβ^52^ and overexpressed in cardiomyocytes of other HF models and failing human hearts, proportionally to severity of HF^53^. Another VO-upregulated protein involved in cell–cell interactions is **nebulin-related anchoring protein** (NRAP), an actin-associated protein localized in intercalated disc, implicated in sarcomere assembly and force transduction. NRAP overexpression in the mouse leads to right ventricular cardiomyopathy^54^. Intermediate filament protein **synemin** (SYNM) that stabilizes intercalated disc and participates in protein kinase A signaling was also upregulated; its absence leads to severe cardiac abnormalities^55^. ACF ventricles showed an upregulation of **microtubule-associated protein 1A** (**MAP1A)**, not previously associated with HF, and upregulation of **major vault protein—**a member of ribonucleoprotein complex relevant for nucleo-cytoplasmatic transport^56^.

*Metabolic genes, ROS and chaperones:* Proteomic analysis confirmed upregulation of glycolytic enzymes and downregulation of FA oxidation seen in targeted PCR analysis. Specifically, ACF ventricles showed upregulation of **pyruvate kinase (PKM**), the final enzyme of glycolysis. Upregulation of fetal isoform (PKM2) was previously observed in failing RV due to PH^57^. In parallel, we observed downregulation of α and β subunits of **trifunctional enzyme** of β-oxidation of fatty acids (**HADHA, HADHB**), more pronounced in volume-overloaded RV than in LV, consistent with switch in myocardial metabolic preference typical to fetal or failing heart^21,39^. Downregulation of mitochondrial 2,4-dienoyl-CoA reductase, an enzyme of β-oxidation of unsaturated fatty acids, was also observed in both VO ventricles. We observed an upregulation of B (fetal) **isoform of creatine kinase (CK-B)** that is typical for failing heart^58^ and a downregulation of its mitochondrial sarcomeric isoform (**CKMT2**), suggesting abnormalities in creatine shuttle and energy transfer.

Both ACF ventricles, but more RV, display strong upregulation of monoaminoxidase-A **(MAOA),** an enzyme that is responsible for degradation of catecholamines^20^. Upregulation of MAOA in ACF ventricles verifies our previous observation and is confirmed also on mRNA level (Fig. 2a) and by western blot (Fig. 5). MAOA might protect myocardium from untoward effects of increased norepinephrine spillover, but it is also a ROS-producing enzyme^59^. If MAO-A upregulation is adaptive or maladaptive in failing myocardium is therefore not known.

Both ACF ventricles showed upregulation of heat shock proteins: **HSPB7** and **HSPB1**. HSPB7 is expressed almost exclusively in striated muscle and is critical for cardiac sarcomere assembly and proteostasis^60^. Genome-wide association study found that variation in HSPB7 locus is associated with reduced LV ejection fraction^61^.

Failing ACF ventricles (more RV than LV) showed upregulated dimethylarginine dimethylhydrolase1 **(DDAH1),** an enzyme that degrades asymmetric dimethyl arginine (ADMA), an endogenous inhibitor of nitric synthase. DDAH1-deficient rats have more severe PH and RV failure if exposed to monocrotaline^62^. In contrast, end-stage HF patients without PH have upregulated myocardial DDAH suggesting a contraregulatory response to putative ADMA elevation and ensuing NO deficit ^63^.

The study has several limitations. To reduce variance, only male rats were studied. Cardiac tolerance to volume overload is worse in males than in females, i.e. changes were more pronounced^64^. Hemodynamics was tested only in resting state, without provocation maneuvers that could discern more subtle changes in cardiac function. Preload changing maneuvers (vena cava balloon inflation) were not performed due to technical reasons, i.e. we cannot report arterial or ventricular elastance values. Proteomic analysis did not detect some proteins that are known to be differentially regulated in HF and were even detected at mRNA level, such as collagens, apelin or apelin receptor. Such discrepancy can be explained by very low expression, insufficient solubility or low molecular weight of the protein with insufficient number of peptides generated by trypsin. To confirm the hypothesis that concomitant PH is responsible for relatively larger RV proteome response to VO, it would be necessary to demonstrate the effect of PH-lowering on RV protein composition, and such experiment was not performed. Changes in pulmonary artery pressure hemodynamics, pulmonary arterioles/venules histology and RV myocardial metabolome in ACF-exposed rats should be further studied to better understand RV-PA coupling in conditions of volume overload.

In conclusion, the study showed that ACF led to changes of molecular markers of heart failure, increased cardiac stress and altered substrate metabolism in both ventricles. RV reacted to ACF relatively more than LV, likely due to larger incremental stroke work due to pulmonary hypertension. Proteomic analysis identified high interventricular concordance of ACF-induced changes, indicating that the RV vs LV differences are explainable hemodynamically, rather than by a presence of “RV-specific” regulatory pathways. Reduction of PA pressure and RV load could therefore be a way how to preserve RV function and prevent adverse remodeling.

## Methods (for details, see supplementary information)

### Heart failure model

Eight-week male Sprague Dawley rats underwent needle ACF/sham operation as described previously^18,65^. After 24 weeks, RV and LV function (echocardiography, biventricular pressure–volume analysis, action potential duration), target gene expression (qPCR) and proteomics (LC–MS/MS) were studied. The study was performed in accordance with relevant guidelines and regulations and was approved by the Animal Ethic Committee of IKEM (#16600/2014-OVZ-30.0-14.3.14). The study was carried out in compliance with the ARRIVE guidelines, if not explicitly stated otherwise.

### Echocardiography and Hemodynamics

Echocardiography was performed under general anesthesia with 10 MHz transducer (Vivid System 7, GE, USA). RV fractional area change (FAC) was defined as difference of end-diastolic and end-systolic RV area, divided by end-diastolic area. RV volumes were calculated using monoplane ellipsoid approximation method^66^. Subsequently, ventricular function was invasively assessed by 2F Pressure–Volume micromanometer-tip catheters (Millar Instruments, Houston, TX, USA) simultaneously introduced into the LV via the right carotid artery and into the RV via the internal jugular vein. Data were analysed by LABCHART PRO software (ADInstruments, Bella Vista, NSW, Australia).

### Contractility and action potential duration measurements

The papillary muscles were dissected from both ventricles. Contraction force and membrane potential was measured as described elsewhere^67^. Action potential duration (APD) was measured at 50% and 90% levels of repolarization (APD50, APD90).

### Gene expression analysis

Samples were taken from RV and LV free wall and placed into RNA later. Total RNA was isolated and genomic DNA removed. RNA quantity and integrity were measured. The RNA was reverse transcribed and qPCR was done using RealTime ready Custom Panel 384–32 (Roche, p.n. 05 582 962 001). The analysis was performed on a LightCycler LC480 (Roche) according to manufacturer’s protocol. Resulting data were analyzed by the ∆Cp method using the R/Bioconductor statistical environment^68–70^. The transcriptional data were normalized to control RV as a chamber with the generally lowest gene expression levels.

### Proteomic analysis

All chemicals were from Sigma-Aldrich, unless stated otherwise. Pooled pulverized myocardial samples (from 7 control and 7 ACF animals) were lysed, digested with trypsin using and labeled with four iTRAQ tags according to the manufacturer´s instructions^71^ (for details see Supplement). The samples were labeled as follows: tags as follows: 114: right ventricle/control, 115: right ventricle/ACF, 116: left ventricle/control, 117: left ventricle/ACF. To remove unbound tags and to pre-fractionate the peptides into four fractions SCX OPTI-TRAP™ Cartridge (Optimized Technologies, OR, USA) was used. Labeled peptide fractions were desalted and dried before LC–MS/MS analysis. 50 cm EASY-Spray column (EASY-Spray column, 50 cm × 75 µm ID, PepMap C18, 2 µm particles, 100 Å pore size) with EASY-Spray™ Source with PepMap100 Pre-column was used for on-line peptide fractionation. A linear gradient was applied for 240 min using Ultimate 3000 Nano LC (Dionex). Data were collected on Thermo Orbitrap Fusion™ in MS^3^ reporter ion quantification mode. The top 10 most intensive peaks from MS^2^ fragmentation were simultaneously selected and fragmented in HCD, MS^3^ masses were acquired in the Orbitrap. In total, three independent iTRAQ analyses using the same pooled myocardial samples were performed. The raw data from all three analyses (each comprising of 4 SCX fractions) were merged and analyzed in Proteome Discoverer 2.2. Data were searched against Rat SwissProt and TrEMBL databases using Sequest HT. FDR 0.01 limit for peptides and proteins was set. Quantitative data were normalized on total peptide amount. Unique and razor peptides were used for quantification.

### Western blot analysis

Pulverized pooled heart samples were lysed, denatured and separated by SDS-PAGE. Proteins were transferred to PVDF membranes, which were than blocked and probed first with primary and then with HRP-conjugated secondary antibodies. The signal was detected using ECL detection system.

### Statistical analysis

Data were assembled and statistically analyzed using JMP 14 software package (SAS, USA). Groups were compared using Student’s t test and Pearson’s correlation coefficient was used for assessment of correlation between continuous variables. The difference of linear regression slope from line of equality (x = y) or between two datasets was tested using GraphPad 6.0. Results are expressed as means ± SD, if not stated otherwise. P-value less than 0.05 was considered significant. Blinding during the data analysis was not considered.

### Supplementary Information

Expanded Materials and Methods section together with additional figures and supplementary proteomic dataset are provided in the supplementary material.

## Supplementary Information

Supplementary Information 1.

Supplementary Information 2.

## Data Availability

All data generated or analyzed during this study are included in this published article (and its Supplementary Information files).
