# Supplementary material for: Right versus left ventricular remodeling in heart failure due to chronic volume overload
Source: Sci Rep. 2021 Aug 24;11:17136. doi: 10.1038/s41598-021-96618-8 (PMC8384875; doi:10.1038/s41598-021-96618-8)
Supplement: Supplementary file 1 — Supplementary Information 1. [file 41598_2021_96618_MOESM1_ESM.pdf]

# SUPPLEMENTAL MATERIAL for ARTICLE

# Right versus left ventricular remodeling in heart failure due to chronic volume overload

*Abbreviated title: Biventricular response to volume overload*

Tereza Havlenova[1,2], Petra Skaroupkova[1], Matus Miklovic[1,2], Matej Behounek[3], Martin Chmel[3], Dagmar Jarkovska[4], Jitka Sviglerova[4], Milan Stengl[4], Michal Kolar[5], Jiri Novotny[5], Jan Benes[1], Ludek Cervenka[1,2], Jiri Petrak[3], Vojtech Melenovsky[1]

Affiliations:

1) Institute for Clinical and Experimental Medicine - IKEM, Prague, Czech Republic
2) Department of Pathophysiology, Second Faculty of Medicine, Charles University, Prague, Czech Republic
3) BIOCEV, First Faculty of Medicine, Charles University, Prague, Czech Republic
4) Department of Physiology, Faculty of Medicine in Pilsen, Charles University, Czech Republic
5) Laboratory of Genomics and Bioinformatics, Institute of Molecular Genetics of the Czech Academy of Sciences, Prague, Czech Republic

## Table of Contents

**Expanded Materials & Methods** ........ **2**
Study Design ........ 2
Echocardiography and Hemodynamics ........ 2
Contractility and action potential duration measurements ........ 3
Gene expression analysis ........ 3
Proteomic analysis and Western blotting ........ 4
Statistics ........ 6
**Figures** ........ **7**
Figure S1A and B: mRNA expression analysis of target genes ........ 8
Figure S2: Myocardial cGMP levels in control and ACF RV and LV. ........ 9
Figure S3: Original western blots used in main manuscript. ........ 10
**References** ........ **11**

# Expanded Materials & Methods

## Study Design

Eight-week old male Sprague Dawley rats (from on-site certified breeding colony at IKEM) weighing 280-320g were randomly distributed into two groups and underwent needle ACF (n=46)/sham (n=20) operation as described previously[1]. Briefly, under general anesthesia induced by intraperitoneal (ip) injection of ketamine/midazolam mixture (50 mg and 5 mg/kg of body weight, respectively), a midline laparotomy was performed and an 18-gauge needle (outer diameter 1.2 mm) was inserted into the infrarenal abdominal aorta and advanced through the vessel wall into the inferior vena cava to create ACF. The needle was removed after temporary clamping the aorta above the site of puncture, which was then sealed with acrylamide tissue glue. The shunt creation was verified by observing pulsatile, bright flow in the inferior vena cava. Sham-operated (control) animals underwent the same procedure without puncture of the vessels.

The rats were kept in air-conditioned animal facility on a 12-/12- h light/dark cycle, were fed standard salt/protein diet (0.45% NaCl, 19–21% protein, SEMED, CR) and had free access to tap water. The rats were weighed weekly and the onset of HF was monitored by scoring as before[1]. Monitored HF signs included piloerection, lethargy, peripheral cyanosis, dyspnea and abdominal swelling (ascites). These parameters were weekly assessed by a single experienced technician and scored 0–3 points (0=absence, 3=maximal presence). HF score was calculated as the sum of points.

All animals that survived till 24 week after surgery were examined (65% survival in ACF group, 95% survival in control group) by at the end of protocol as described below. The presence of ACF was verified from laparotomy (animals with failed ACF would be excluded, but such situation did not happen) and the animals were exsanguinated. The coronary tree of the excised heart was rapidly flushed with cardioplegic solution. The organs were weighed and normalized to body weight. The investigation conformed to the NIH Guide for the care and use of laboratory animals (NIH Publication No. 85-23, 1996), Animal protection laws of the Czech Republic (311/1997) and was approved by the Animal Ethic Committee of IKEM (#16600/2014-OVZ-30.0-14.3.14). The study was carried out in compliance with the ARRIVE guidelines, if not explicitly stated otherwise.

## Echocardiography and Hemodynamics

Echocardiography (available from all animals) was performed under general anesthesia (ketamine/midazolam mixture i.p. as described above) with 10 MHz transducer (Vivid System 7, GE, USA) 24th week post-ACF creation. End-systolic (ESV) and end-diastolic (EDV) LV volumes were derived by cubic equation and stroke volume (SV) as their difference. Cardiac output (CO) was calculated as product of stroke volume times heart rate (HR). Relative LV wall thickness was defined as sum of end-diastolic interventricular septum and posterior wall thickness, divided by end-diastolic LV diameter. LV fractional shortening (FS) was calculated as difference of end-diastolic and end-systolic LV diameter, divided by end-diastolic diameter. RV fractional area change (FAC) was defined as difference of end-

diastolic and end-systolic RV area, divided by end-diastolic area. RV volumes were calculated using monoplane ellipsoid approximation method[2]. 30 ACF animals and 19 control animals were analyzed by echocardiography.

Subsequently, rats were intubated with a plastic cannula, relaxed with pancuronium (Pavulon, 0.16 mg/kg, N.V. Organon, Oss, Netherlands) and artificially ventilated (rodent ventilator; Ugo Basile, Gemonio VA, Italy, FiO2 = 21%). Vagal blockade (atropine 0.10 mg/kg) was administered to prevent interfering reflexes. LV function was invasively assessed by 2F Pressure–Volume (P–V) micromanometer-tip catheter (Millar Instruments, Houston, TX, USA) introduced into the LV cavity via the right carotid artery. Simultaneously, another 2F PV catheter was introduced into the right ventricle via the internal jugular vein to study RV function. The volume signals were calibrated by end-diastolic and end-systolic volumes obtained by echocardiography shortly before invasive recordings as done before[3]. Data were acquired using an 8-channel Power Lab recorder and were analysed by LABCHART PRO software (ADInstruments, Bella Vista, NSW, Australia). Chamber wall stress was calculated as (peak chamber pressure x chamber end-diastolic volume)/chamber mass obtained by direct weighting[4]. Usable hemodynamic pressure volume recordings were obtained from 26 ACF animals and from 16 sham-operated control animals.

**Contractility and action potential duration measurements**

The studies were conducted as described elsewhere[5]. The papillary muscles were dissected from both ventricles and placed into an experimental chamber. The preparation was perfused with 36°C warm, oxygenated Tyrode solution at a constant flow rate (6–10 mL/min). After a stabilization period (30 min), the preparation was stimulated at various frequencies (0.5, 1, 2, 3, and 5 Hz; Pulsemaster Multi-Channel Stimulator A300, World Precision Instruments, Inc., FL, USA). Contraction force was measured by an isometric force transducer (model F30; Hugo Sachs Electronik - Harvard Apparatus, GmBH, Germany) and membrane potential was acquired using glass microelectrodes filled with 3M KCl (resistance >20 MΩ; Microelectrode Puller P-1000, Sutter Instrument, CA, USA). APD was measured at 50% and 90% levels of repolarization (APD50, APD90). Data were recorded and analyzed using the National Instruments data acquisition hardware and software (National Instruments, Austin, TX, USA).

**Gene expression analysis**

Samples were taken from RV and LV free wall and placed into RNAlater. Total RNA was isolated by RNeasy Micro Kit (Qiagen) according to the procedure for fibrous tissues (cardiomyocytes). All extracts were treated by DNase I (Qiagen) to remove contaminating genomic DNA. The quantity of the RNA was measured on a NanoDrop ND-1000 (NanoDrop Technologies LLC). RNA integrity was assessed on Agilent 2100 Bioanalyser (Agilent Technologies). All RNA samples had RNA integrity number RIN > 7. The RNA (1000 ng) was reverse transcribed using QuantiTect Reverse Transcription Kit (Qiagen). Either 1.5 ng or 5 ng of cDNA was used in the second step of RT-qPCR using RealTime

ready Custom Panel 384–32 (Roche, p.n. 05 582 962 001) containing function tested pre-plated qPCR assays for 29 target genes (*Acadm, Acta1, Angpt1, Angpt2, Apln, Aplnr, Atp2a2, Cs, Gucy1a3, Hif1a, Hk1, Il6, Maoa, Myh6, Myh7, Nos1, Nos2, Nos3, Nppa, Npr1, Npr2, Pde5a, Pde9a, Pkg Slc2a1, Slc2a4, Tgm2, Tnfrsf1a, Vegfa*) and 3 reference genes (*Hprt1, Sdha, Tbp*). Each assay included primers and a short FAM-labelled hydrolysis probe containing locked nucleic acid. The kit consisted of ten 384 well plates that contained inter-plate calibrator samples. The protocol was performed on a LightCycler LC480 (Roche) instrument according to manufacturer's protocol.

The resulting data were analyzed using the ΔC_p method[6] within the R/Bioconductor statistical environment[7]. The inter-plate calibration was performed as follows: within each plate and for each target, C_p of calibrator samples were subtracted from C_p of biological samples and mean C_p of calibrator samples from all plates was added. Next, missing C_p values (less than 1 % of all values) were imputed using the non-detects package[8]. Finally, the mean of three stable reference genes (*Hprt1, Tbp and Sdha*) was subtracted to obtain 〖ΔC〗 p values.

**Proteomic analysis and Western blotting**

*Myocardial sample preparation:* All chemicals were from Sigma-Aldrich, unless stated otherwise. Myocardial samples from both ventricles were pulverized using mortar and pestle under liquid nitrogen. Pooled "Control" and "ACF" samples were then prepared by mixing equal amounts (10 mg) of pulverized material from each animal in the respective cohort (n=7). Pooled samples were lysed in 1 mL of lysis buffer (8.4 M urea, 50 mM DTT, 5 % CHAPS) for 30 minutes at room temperature. The lysates were sedimented at 20,000-× g for 20 minutes at 22 °C. The supernatants were collected, and total protein concentration was determined with Bio-Rad Protein Assay Dye Reagent Concentrate (Bio-Rad, CA).

*Filter assisted sample preparation (FASP) and iTRAQ labeling:* Protein lysates were processed by the FASP method[9]. Lysates of pooled samples (120 $\mu$g) were diluted with ddH2O to lower the urea concentration to 7 M and transferred to 30 kDa cut-off filters (Microcon® 30 kDa Centrifugal Filter Unit with Ultracel® 30 membrane) and centrifuged at 7,000-× g for 5 minutes. The filters were than washed 2× with 400 $\mu$l urea buffer (8 M urea in 60 mM HEPES, pH 8.2) and 3× with 400 $\mu$l 60 mM HEPES, pH 8.2, at 10,000-× g for 15 minutes. Labelling was performed exactly according the manufacturers protocol (iTRAQ® Reagents Multiplex Kit, SCIEX). Briefly, 20 $\mu$l dissolution buffer (TEAB), 1 $\mu$l denaturant (SDS), and 2 $\mu$l reducing reagent (TCEP) were added to the samples, and the filters were incubated at 22 °C for 1 hour with gentle shaking. Samples were then alkylated by 1 $\mu$l of cysteine-blocking reagent (MMTS), and the incubated at room temperature for 10 minutes. Digestion with 11 $\mu$l (0.8 $\mu$g/$\mu$l) of trypsin solution (Promega, Sequencing grade modified trypsin) was performed overnight at 37 °C. The samples were labelled with four isobaric tags as follows: 114 – right ventricle/control, 115 - right ventricle/ ACF, 116 – left ventricle/ control, 117 – left ventricle/ ACF. The iTRAQ reagents dissolved in ethanol were transferred to each sample (still in the filter) and incubated for 1 hour at room temperature

with gentle shaking. After incubation, the filters were centrifuged at 8,000-× g for 5 minutes and washed twice with 50 $\mu$l ddH2O and once with 20 $\mu$l 0.5 M NaCl at 8,000-× g for 5 minutes. The four flow-through fractions were pooled into one tube, and dried in Concentrator plus (Eppendorf Concentrator Plus 5305).

*Strong cation exchange chromatography and desalting:* To remove unbound tags and to pre-fractionate the peptides before LC-MS/MS, manually operated SCX OPTI-TRAP™ Cartridge (Optimized Technologies, OR, USA) was used. Dried iTRAQ-labeled sample were solubilized in 1 mL SCX-LOAD buffer (5 mM KH2PO4, pH 3 in 25% MeCN) in an ultrasonic bath for 10 minutes. The cartridge was washed with 500 $\mu$l SCX-CLEAN buffer (350 mM KCl in SCX-LOAD buffer) and equilibrated with 500 $\mu$l SCX-LOAD buffer. Samples were loaded to the column and washed with 600 $\mu$l SCX-LOAD. Peptides were eluted into four fractions with 200 $\mu$l SCX-LOAD buffer supplemented with of 50, 100, 200, and 350 mM KCl. The eluted peptides were vacuum-dried. Desalting was performed using manually operated OPTI-TRAP Peptide Concentration & Desalting cartridge (Optimized Technologies, OR, USA). Dried samples were dissolved in in 30 µl of 80 % MeCN with 0.1% TFA and sonicated for 10 minutes and then diluted by 0.1 % TFA to a final volume of 300 µl. The cartridge was washed with 700 µl 80 % MeCN with 0.1 % TFA, and equilibrated with 600 µl 0.1 % TFA. Samples were then loaded, washed with 500 µl 0.1 % TFA, eluted with 200 µl 80 % ACN with 0.1 % TFA, and vacuum –dried (Eppendorf Concentrator Plus 5305).

*LC-MS/MS analysis:* Samples were solubilized and loaded to 50 cm EASY-Spray column (EASY-Spray column, 50 cm x 75 µm ID, PepMap C18, 2 µm particles, 100 Å pore size) with EASY-Spray™ Source with PepMap100 Pre-column (5mm x 300µm C18, 2 µm particles 100 Å). A linear gradient from 4 % B to 35 % B (A: water 0.1 % FA, B: MeCN 0.1 % FA) was applied for 240 minutes at 300 nl/min using Ultimate 3000 nano LC (Dionex). Data were collected on Thermo Orbitrap Fusion™ in $MS^3$ reporter ion quantification mode. $MS^1$ were collected every 4 seconds at resolution 120K, the maximum possible number of precursors were selected for $MS^2$ CID fragmentation and detection in ion trap (injection time 60 ms). The top 10 most intensive peaks from $MS^2$ fragmentation were simultaneously selected and fragmented in HCD. $MS^3$ masses were acquired in Orbitrap at resolution 60K (injection time 140 ms). In total, we performed three independent iTRAQ analyses using the same pooled myocardial samples. The raw data from all three analyses (including 4 SCX fractions each) were merged and analyzed in Proteome Discoverer 2.2. Tolerances were sets: 10 ppm for precursor, 0.6 Da for $MS^2$ fragments, and 20 ppm for $MS^3$ quantification fragments. Appropriate iTRAQ modifications (+144.102) were set as stable (N term and K) and variable (Y). MethylThio (+45,988) modification was set as stable on C. M oxidation (+15.995) was set as a dynamic modification. The search engine was Sequest HT. Data were searched against 2020, May version of SwissProt (Rat, Reviewed – 8,110 entries) and TrEMBL (Rat, Unreviewed – 29,953 entries). Percolator was used for FDR estimation, and 1 % FDRs limits for peptides

and proteins were used. Quantification data were normalized on total peptide amount. Unique and razor peptides were used for quantification.

*Western blot analysis:* Pulverized pooled heart samples were lysed in lysis buffer (RIPA buffer, Sigma-Aldrich) for 30 minutes in 4 °C and sedimented at 20,000-× g for 20 minutes at 4 °C. The supernatants were collected, and the total protein concentration were determined by BCA assay (Bicinchoninic Acid Kit, Sigma-Aldrich) using Nanodrop One (ThermoFisher). Protein samples (40 $\mu$g) were denatured at 100 °C for 10 minutes using sample buffer (60 mM Tris–HCL pH 6.8, 2 % SDS, 10 % glycerol, 0.02 % bromphenol blue) and separated by SDS-PAGE. Proteins were transferred to PVDF membranes and blocked at room temperature for 30 minutes in 5 % nonfat dry milk in PBS-T buffer (Dulbecco's Phosphate Buffered Saline, with 1 % Tween-20). Membranes were washed 3 times with PBS-T buffer and then incubated with a primary antibody at room temperature for 2 hours (Annexin V, Abcam, ab141961:1,000; MAO-A, Santa Cruz, sc-271123 - 1:300; NCAM1, Abcam, ab95153, 1:400; Periostin, Abcam, ab92460, 1:1000; TGM2, Millipore, JBC1863304, 1:400). The membranes were washed 3 times with PBS-T buffer and then incubated with an appropriate secondary HRP-conjugated antibody diluted 1:10 000 (Anti-Rb IgG Jackson ImmunoResearch, 711-165-152; Anti-Mouse IgG Santa Cruz, sc-2005, 1:10,000 or Anti-Goat IgG Santa Cruz, sc-2354, 1:10,000) at room temperature for 30 minutes. The membranes were then washed 3 times with PBS-T buffer and incubated with an ECL detection system (Western Blotting Luminol Reagent, Santa Cruz Biotechnology, sc-2048) at room temperature for 3 minutes. Chemiluminescence was captured by ChemiDoc™ MP System equipped with Image Lab™ software 5.2.1 (Bio-rad). Presented images were compliant with the digital image and integrity policies of the journal.

**Statistics**

Data were assembled and statistically analyzed using JMP 14 software package (SAS, USA). Groups were compared using Student's t test and Pearson's correlation coefficient was used for assessment of correlation between continuous variables. Results are expressed as means±SD. P-value less than 0.05 was considered significant.

# Figures and Tables

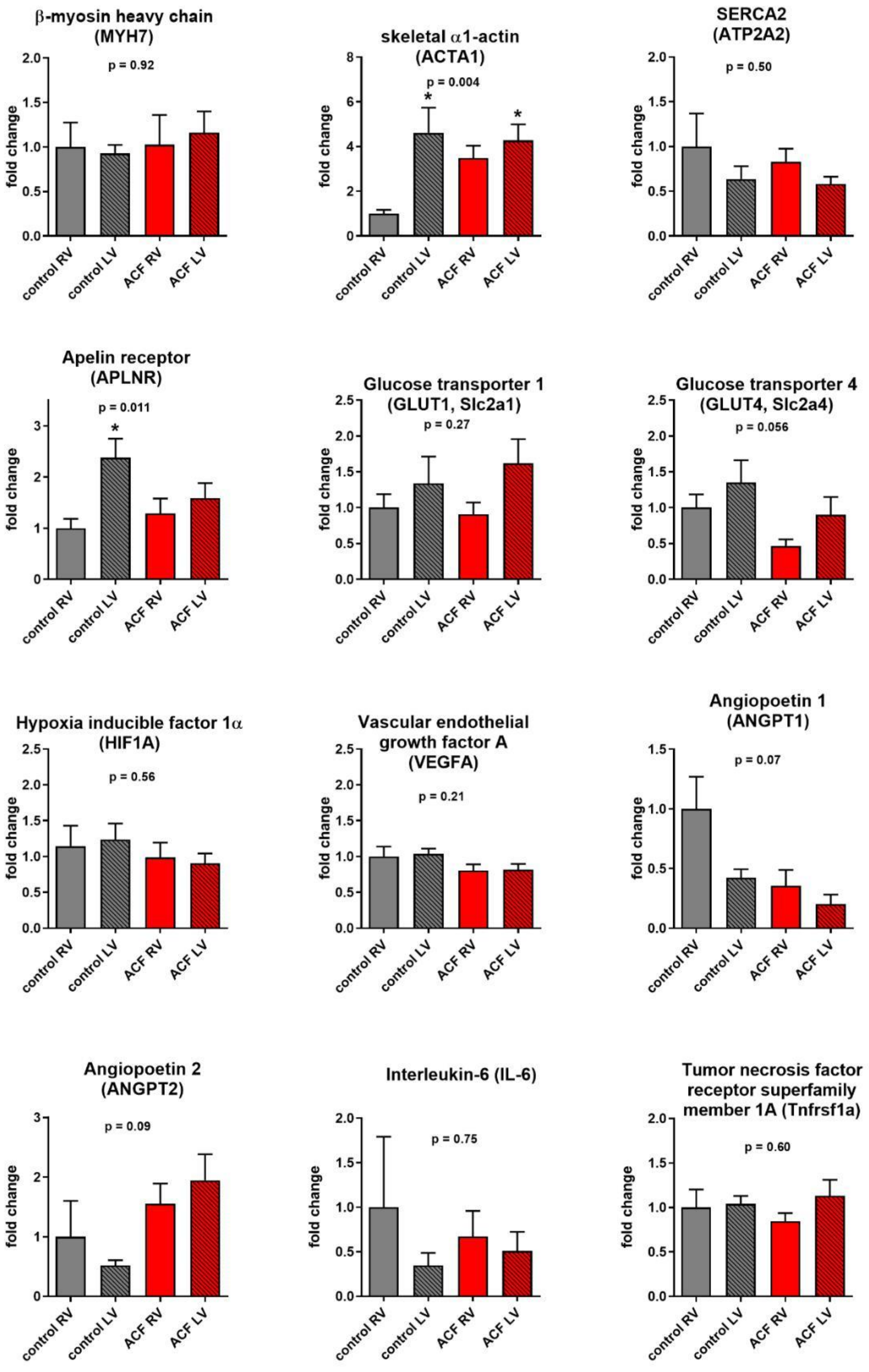

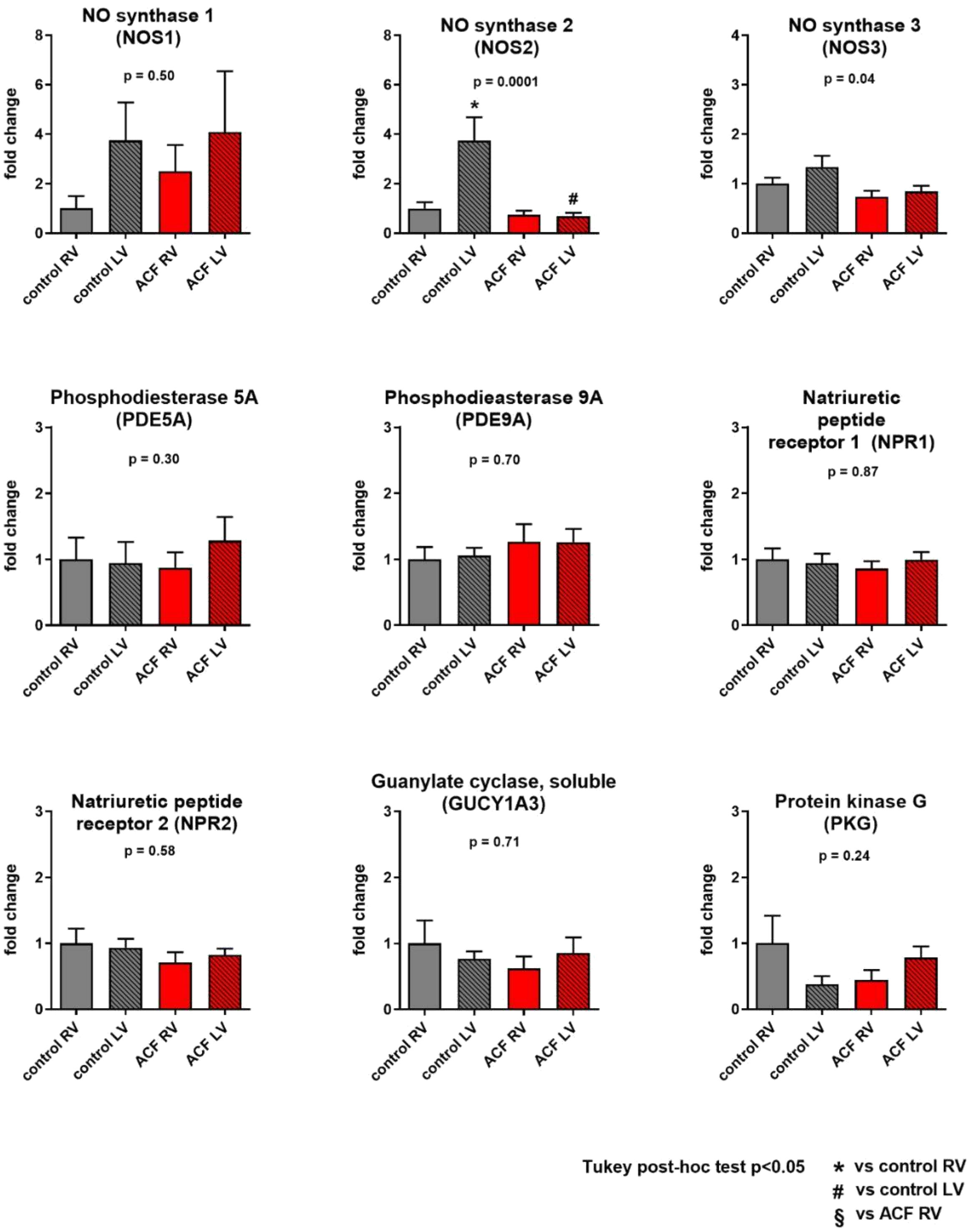


**Figure S1A and B: mRNA expression analysis of target genes.**

Gene mRNA expression analysis of 21 target genes. Data are presented as means ± S.E.M. Changes normalized to control RV. N=12 in each group. P value: ANOVA, and Tukey post-hoc tests.

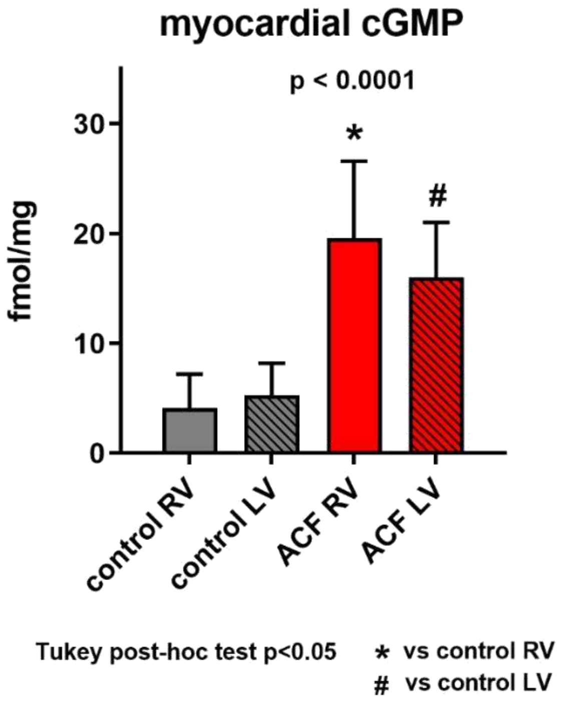


**Figure S2: Myocardial cGMP levels in control and ACF RV and LV.**

Data are presented as means ± S.E.M. N=8 in each group. P value: ANOVA, and Tukey post-hoc tests.

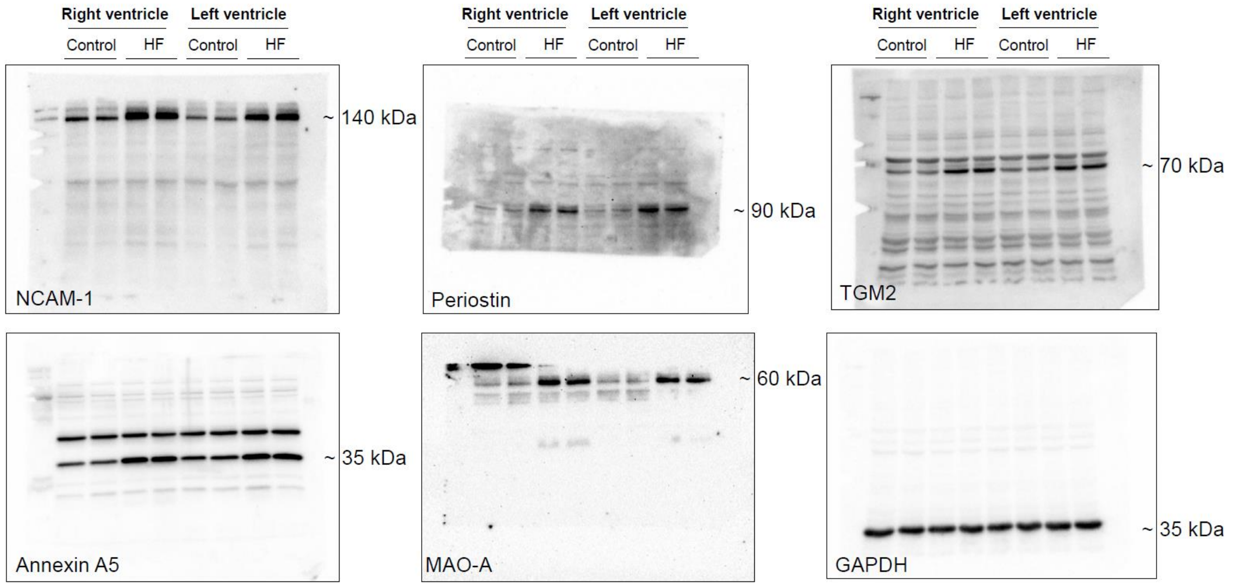


**Figure S3: Original western blots used in main manuscript.**

Western blots were prepared and developed as described in the method section.

# References


1. Melenovsky, V. *et al.* The Course of Heart Failure Development and Mortality in Rats with Volume Overload due to Aorto-Caval Fistula. *KBR* **35**, 167–173 (2012).
2. Lange, P. E. *et al.* Value of image enhancement and injection of contrast medium for right ventricular volume determination by two-dimensional echocardiography in congenital heart disease. *The American Journal of Cardiology* **55**, 152–157 (1985).
3. Červenka, L. *et al.* Inhibition of soluble epoxide hydrolase counteracts the development of renal dysfunction and progression of congestive heart failure in Ren-2 transgenic hypertensive rats with aorto-caval fistula. *Clinical and Experimental Pharmacology and Physiology* **42**, 795–807 (2015).
4. Bartelds, B. *et al.* Differential responses of the right ventricle to abnormal loading conditions in mice: pressure vs. volume load. *European Journal of Heart Failure* **13**, 1275–1282 (2011).
5. Švíglerová, J. *et al.* Cardiac remodeling in rats with renal failure shows interventricular differences. *Exp Biol Med (Maywood)* **237**, 1056–1067 (2012).
6. Livak, K. J. & Schmittgen, T. D. Analysis of Relative Gene Expression Data Using Real-Time Quantitative PCR and the 2−ΔΔCT Method. *Methods* **25**, 402–408 (2001).
7. Huber, W. *et al.* Orchestrating high-throughput genomic analysis with Bioconductor. *Nature Methods* **12**, 115–121 (2015).
8. McCall, M. N., McMurray, H. R., Land, H. & Almudevar, A. On non-detects in qPCR data. *Bioinformatics* **30**, 2310–2316 (2014).
9. Wiśniewski, J. R., Zougman, A., Nagaraj, N. & Mann, M. Universal sample preparation method for proteome analysis. *Nature Methods* **6**, 359–362 (2009).
